# Ethical dilemmas in psychiatry through the lens of equity and human rights

**DOI:** 10.1192/j.eurpsy.2026.10357

**Published:** 2026-07-31

**Authors:** M. Schouler-Ocak

**Affiliations:** Psychiatric Univesity Clinic of Charité at St. Hedwig.-Hospital, Charité – Universitätsmedizin Berlin, corporate member of Freie Universität Berlin and Humboldt-Universität zu Berlin, Berlin, Germany

## Abstract

**Abstract:**

Ethical dilemmas are a central and everyday challenge in psychiatric practice, particularly where questions of equity and human rights are concerned. Psychiatry operates in a unique space between care, social control, and legal authority, which makes ethical tensions especially visible and, at times, unavoidable. This talk examines key ethical dilemmas in psychiatry through the lens of equity and human rights, focusing on issues such as involuntary treatment, coercive practices, decision-making capacity, stigma, and unequal access to mental health care. It highlights how social and structural inequalities—related to socioeconomic status, ethnicity, gender, migration status, disability, and other forms of marginalization—shape both mental health outcomes and patients’ experiences within psychiatric systems. Drawing on international human rights frameworks, including the United Nations Convention on the Rights of Persons with Disabilities, the presentation explores tensions between autonomy, beneficence, non-maleficence, and justice in everyday clinical practice. By integrating ethical reflection with real-world clinical challenges, the talk aims to stimulate discussion on how rights-based, person-centered approaches can help reduce harm, enhance dignity, and promote more equitable and humane psychiatric care.

**Disclosure of Interest:**

None Declared

